# miR−21 and NT-proBNP Correlate with Echocardiographic Parameters of Atrial Dysfunction and Predict Atrial Fibrillation

**DOI:** 10.3390/jcm9041118

**Published:** 2020-04-14

**Authors:** Jan-Thorben Sieweke, Tobias Jonathan Pfeffer, Saskia Biber, Shambhabi Chatterjee, Karin Weissenborn, Gerrit M. Grosse, Jan Hagemus, Anselm A. Derda, Dominik Berliner, Ralf Lichtinghagen, Denise Hilfiker-Kleiner, Johann Bauersachs, Christian Bär, Thomas Thum, Udo Bavendiek

**Affiliations:** 1 Department of Cardiology and Angiology, Hannover Medical School, 30625 Hannover, Germany; sieweke.jan-thorben@mh-hannover.de (J.-T.S.); Pfeffer.Tobias.J@mh-hannover.de (T.J.P.); Saskia.Biber@stud.mh-hannover.de (S.B.); Jan@Hagemus.com (J.H.); Derda.Anselm@mh-hannover.de (A.A.D.); Berliner.Dominik@mh-hannover.de (D.B.); hilfiker.denise@mh-hannover.de (D.H.K.); Bauersachs.Johann@mh-hannover.de (J.B.); 2 Institute of Molecular and Translational Therapeutic Strategies and Center for Translational Regenerative Medicine, Hannover Medical School, 30625 Hannover, Germany; Shambhabi.Chatterjee@stud.mh-hannover.de (S.C.); baer.christian@mh-hannover.de (C.B.); thum.thomas@mh-hannover.de (T.T.); 3 Department of Neurology, Hannover Medical School, 30625 Hannover, Germany; Weissenborn.Karin@mh-hannover.de (K.W.); grosse.gerrit@mh-hannover.de (G.M.G.); 4 Department of Clinical Chemistry, Hannover Medical School, 30625 Hannover, Germany; Lichtinghagen.Ralf@mh-hannover.de

**Keywords:** Atrial fibrillation, biomarker, echocardiography, microRNA, NT-proBNP

## Abstract

This study aimed to investigate the association of circulating biomarkers with echocardiographic parameters of atrial remodelling and their potential for predicting atrial fibrillation (AF). In patients with and without AF (*n* = 21 and *n* = 60) the following serum biomarkers were determined: soluble ST2 (sST2), Galectin−3 (Gal-3), N-terminal pro-brain natriuretic peptide (NT-proBNP), microRNA (miR)−21, −29a, −133a, −146b and −328. Comprehensive transthoracic echocardiography was performed in all participants. Biomarkers were significantly altered in patients with AF. The echocardiographic parameter septal PA-TDI, indicating left atrial (LA) remodelling, correlated with concentrations of sST2 (r = 0.249, *p* = 0.048), miR−21 (r = −0.277, *p* = 0.012), miR−29a (r = −0.269, *p* = 0.015), miR−146b (r = −0.319, *p* = 0.004) and miR−328 (r = −0.296, *p* = 0.008). In particular, NT-proBNP showed a strong correlation with echocardiographic markers of LA remodelling and dysfunction (septal PA-TDI: r = 0.444, *p* < 0.001, LAVI/a’: r = 0.457, *p* = 0.001, SRa: r = 0.581, *p* < 0.001). Multivariate Cox regressions analysis highlighted miR−21 and NT-proBNP as predictive markers for AF (miR−21: hazard ratio (HR) 0.16; 95% confidence interval (CI) 0.04–0.7, *p* = 0.009; NT-proBNP: HR 1.002 95%CI 1.001–1.004, *p* = 0.006). Combination of NT-proBNP and miR−21 had the best accuracy to discriminate patients with AF from those without AF (area under the curve (AUC)= 0.843). Our findings indicate that miR−21 and NT-proBNP correlate with echocardiographic parameters of atrial remodeling and predict AF, in particular if combined.

## 1. Introduction

Atrial fibrillation (AF) is one of the main causes of stroke, particular in the elderly [1]. Stroke represents one of the most common reasons for death, disability and incapacity for work in western countries. Therefore, there is very large need for early detection of AF to prevent cardio-embolic stroke and this has become a research focus recently [2]. Diagnosis of AF is challenging as AF often occurs without any clinical symptoms, leading to a delay of diagnosis and delayed therapeutic anticoagulation in patients at high risk for cardio-embolic events. 

Therefore, studies discussed prolongation of electrocardiogram (ECG) monitoring in patients after stroke even beyond 72 h as recommended by the European Society of Cardiology guidelines [3,4,5]. However, extended ECG monitoring is resource consuming and potentially compromises patients [6,7,8,9]. Therefore, it would be of interest to identify predictors indicating patients at high risk for AF which can selectively be assigned to extended ECG-monitoring.

It is well known that left atrial (LA) remodeling, dysfunction and fibrosis contribute to the onset and maintenance of AF [10,11]. As echocardiography is an important imaging tool to evaluate parameters of LA function, remodeling and fibrosis in patients with AF, echocardiography might contribute to early diagnosis of AF [10,12,13,14,15]. Recently, we identified septal total atrial conduction time (PA-TDI interval) as a predictor of AF in patients in sinus rhythm [16]. Furthermore, the ratio of LA volume index to tissue Doppler a′ (LAVI/a′) and the second negative peak strain rate during LA contraction (SRa) assessed by speckle tracking echocardiography, correlate with LA remodeling and fibrosis and are predictors of AF [11,14,16]. 

N-terminal fragment of pro-B-type natriuretic peptide (NT-proBNP), Galectin−3 (Gal3) and soluble suppression of tumorigenicity 2 (sST2), are associated with myocardial fibrosis [17,18,19] and have a predictive value for an adverse outcome in heart failure patients [20,21]. Gal3 levels are associated with the incident of AF and correlate with atrial fibrosis [22,23], BNP and sST2 are associated with AF reccurence [24]. 

MicroRNAs (miRs) comprise endogenous small non-coding RNA molecules, which negatively regulate gene/protein expression post-transcriptionally by complementary binding of messenger RNA-targets, thereby stabilizing them or blocking their translation [25]. miRs are emerging as therapeutic targets in cardiovascular diseases [26,27,28]. In addition, since they are stable and readily detectable in the bloodstream, miRs represent promising biomarkers for early detection of cardiac diseases. In previous studies, evidence was provided for miRs to be associated with atrial remodeling, atrial fibrosis and atrial fibrillation [29,30,31].

Hence, the present study investigated whether different biomarkers and miRs associated with LA remodeling and fibrosis correlate with echocardiographic parameters of LA function and remodeling, and could serve as predictive biomarkers for the detection of subclinical AF. 

## 2. Methods

### 2.1. Study Design and Participants

The present study is a prospective, semi-blinded, single center controlled study, which was approved by the local ethics committee of Hannover Medical School (application number: 3316–2016). The study complies with the Declaration of Helsinki and all participants gave written informed consent. This study is a sub-study of a recent published manuscript describing prediction of AF by echocardiographic parameters in patients after embolic stroke of undetermined source and control cohorts, which gives further detailed information about in-/exclusion criteria and clinical work-flow [16]. Subsequently, included participants were categorized into cohorts and groups: (1) Control cohort: young participants without AF, old participants without AF, and patients with acute ischemic stroke without AF. (2) AF-cohort: patients with paroxysmal AF, patients with stroke and new-onset of AF. All participants underwent 12-lead ECG, and detailed transthoracic echocardiography. 

### 2.2. Echocardiography

**Figure 1:**
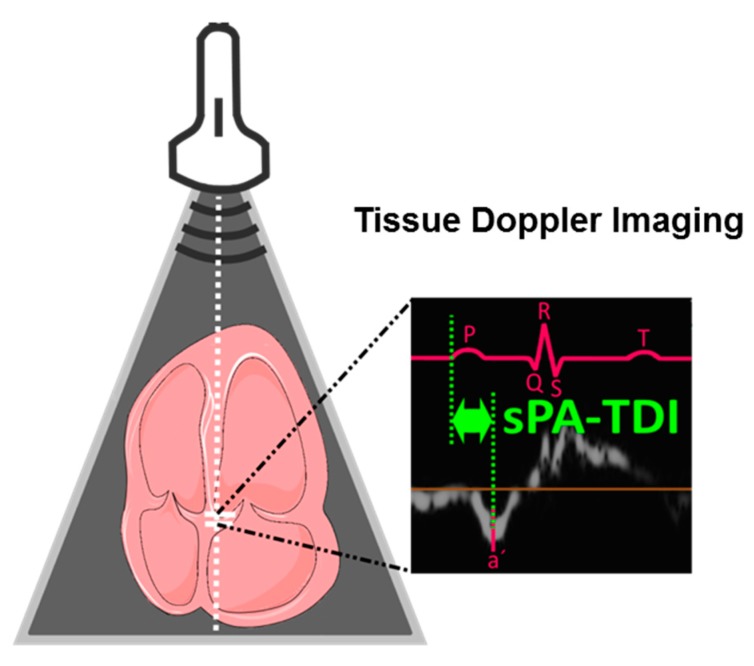
Determination of septal PA-TDI (sPA-TDI). Septal PA-TDI was determined in tissue Doppler imaging and defined as interval between the onset of P-wave in lead II of the electrocardiogram (ECG) and the peak a′-wave of the septal mitral valve annulus.

Data of transthoracic echocardiography were collected prospectively according to the American Society of Echocardiography guidelines [32]. Echocardiographic parameters of LA function and remodeling were determined as recently described [16]. In brief, dedicated acquisitions from the apical approach were performed for correct assessment of LA volumes according to recent recommendations of the American Society of Echocardiography (ASE) and the European Association of Cardiovascular Imaging (EACVI) [33]. Left atrial volume was determined by biplane area length method in apical 4– and 2– chamber views at the ventricular end-systole. Subsequently, left atrial volume was indexed to surface area. In tissue Doppler imaging septal and lateral late diastolic peak tissue Doppler velocity (a′) was determined. The assessment of LAVI/a′ was performed with the average of septal and lateral a′. The interval between the onset of *p*-wave in lead II of the ECG on echocardiographic images and the peak a′-wave of the septal or lateral mitral valve (MV) annulus in tissue Doppler imaging was defined as PA-TDI septal or lateral and is illustrated for PA-TDI septal in Figure 1. Of note, only patients in sinus rhythm during echocardiographic examination were included in this study as parameters for LA remodeling cannot be determined in the presence of AF. Echocardiography was performed by one expert investigator, who was unaware of patients’ diagnosis and results of biomarker and microRNA-testing.

### 2.3. Holter Electrocardiogram (ECG) Monitoring

Management of Holter ECG-Monitoring was previously described [16]. In brief, all participants had a 12-channel surface ECG. Participants without stroke were included in the control group, if there was no history of AF and/or no documented AF in a 24 h-long-term-ECG. In patients after a stroke, a Holter-ECG-monitoring was scheduled for 72 h. All ECG recordings were analyzed by two independent and blinded professionals applying current guidelines on atrial fibrillation [34].

### 2.4. Biomarker Assays

Plasma and serum samples were collected after echocardiographic examination. RNase/DNase clean aliquots of ethylenediaminetetraacetate (EDTA) plasma and serum were stored at −80 °C. Levels of NT-proBNP were determined in EDTA plasma at the Department of Clinical Chemistry at Hannover Medical School. The measurement of plasma Gal3 (Human Galectin−3 Platinum Elisa, eBiosciences, San Diego, CA, USA) and sST2 (Presage^®^ ST2 Assay, Critical Diagnostics, San Diego, CA, USA) were conducted according to the manufacturer’s instructions. 

### 2.5. RNA Isolation from EDTA Plasma

Purification and isolation of total RNA from 150 µL plasma was performed using miRNeasyMiniKit (QIAGEN, Venlo, Netherlands) according to the manufacturer’s instructions. Caenorhabditis elegans (cel miR−39, 3.5 µL 0.267 pmol/µL) as spiked-in RNA was added before starting the isolation procedure. 10 μL of RNA-solution was obtained for further analysis. 

### 2.6. Real-Time Polymerase Chain Reaction (PCR)-Based Amplification of MicroRNAs

A TaqMan MicroRNA Reverse Transcription Kit (Applied Biosystems) was used according to the manufacturer’s protocol to transcribe isolated RNA to complementary DNA (cDNA). HSA-miR−21–5p, hsa-miR−29a−3p, hsa-miR−133a−3p, hsa-miR−146b−5p, hsa-miR−328−3p, hsa-miR−486−5p (endogenous control) and cel-miR−39−3p primers (spiked-in control) were used (Primer sequences of miRs are shown in Table 1). Subsequently, the defined miRs were amplified with quantitative real-time polymerase chain reaction (qRT-PCR) utilizing specific TaqMan MicroRNA assays (Applied Biosystems). Plasma levels of the described miRs were normalized using the following miRs: 1. Synthetic *Caenorhabditis elegans* miR−39 (cel MiR−39) as spiked-in RNA as external control and qPCR normalizer [35,36], 2. Endogenous, stably expressed and abundant in plasma miR−486 [37,38,39] as previously described.

### 2.7. Statistical Analysis

Statistical analysis and graphical presentation were performed using SPSS Statistics 25 (IBM SPSS Statistics 25) and GraphPad Prism 7.04 (Graph Pad Software, San Diego, CA, USA). Categorical variables are given as *n* (%). Continuous variables are presented as mean ± standard deviation (SD) for quantification of normally distributed variables, or median and interquartile ranges (IQR) for non-normally distributed variables. Normality and variance homogeneity were checked by Shapiro–Wilk and D’Agostino Pearson tests. Comparison between the groups was performed using the Student’s *t*-test for Gaussian distributed data and the Mann–Whitney test in non-normally distributed data. Analysis of variance (ANOVA) was performed followed by a Bonferroni test or Dunn’s test for multiple comparisons, respectively. Categorical variables were evaluated by the Chi-square test. To compare correlation with biomarkers, the Spearman’s rank order correlations was used.

All patients (*n* = 81) were stratified to two categories in a random manner: training set *n* = 35 and test set *n* = 46. The training set was analyzed to build a miR prediction model that was later confirmed in test and entire sets. In the training set, we screened out miRs with a significant *p*-value of less than 0.1 indicating potential association with AF (s-ST2, Gal3, miR−21, miR−29a, miR−133a, miR−146b, miR−328, NT-proBNP) by using univariate logistic regression (provided in Table S1). Thereafter, this signature was validated in the test set and the entire set. receiver operating characteristic (ROC) analysis was also carried out to validate accuracy and feasibility of the miR model (presented in Table S2). Subsequently predictors of AF were determined using a stepwise multivariate logistic regression analysis with variables, which significantly linked to AF in univariate analysis (*p* < 0.05; Gal3, miR−21, miR−29a, miR−146b, miR−328, NT-proBNP). Results from the regression analyses are presented as hazard ratios (HRs) with 95% confidence intervals (CIs).

The discriminative ability of the risk prediction model was assessed by the area under the ROC curve. Youden’s index was determined to ascertain cut-off values of the variables independently associated with AF. Subsequently, under the assumption of a dichotomous distribution using cut-off values, biomarkers and miRs independently predicting AF were compared by a McNemar test (presented in Table S3). A two-sided *p*-value of <0.05 was considered statistically significant.

## 3. Results

### 3.1. Patient Characteristics

In a recently published pilot-study with the aim to identify echocardiographic parameters predicting AF in patients with embolic stroke of undetermined source and controls between 1 August 2016 and 30 April 2017 a total of 175 patients were included [16]. In the present sub-study, we investigated 81 patients who approved additional biomarker analysis with the intention to predict AF and correlate results of the biomarker analysis with echocardiographic parameters associated with LA function and remodeling. 

**Figure 2:**
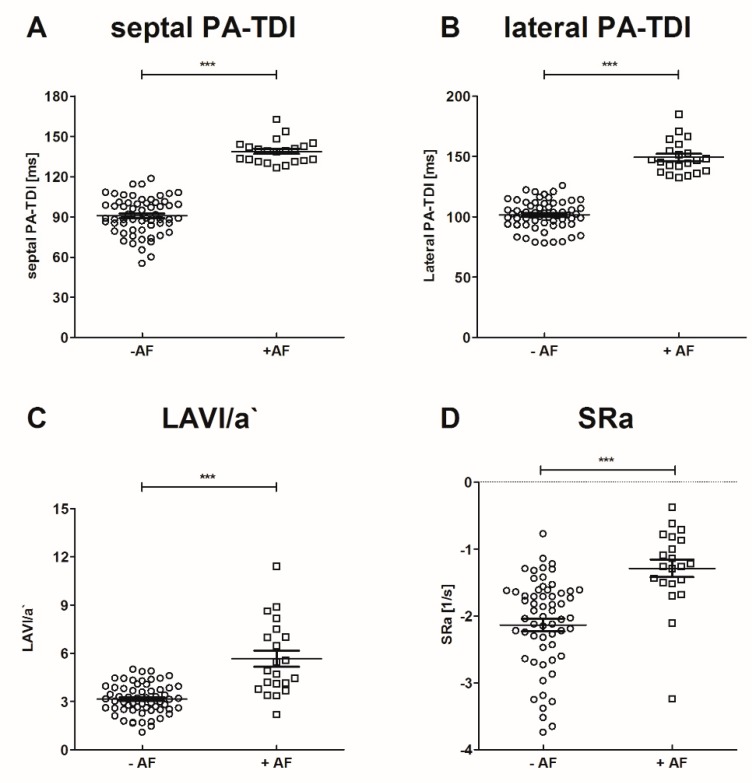
Echocardiographic parameters of left atrial (LA) function in patients with and without AF. *** *p* < 0.001.

Participants were classified according to previously diagnosed AF and/or stroke into the following groups: young participants without stroke and without AF (*n* = 13), old participants without AF and without stroke (*n* = 10), patients with acute ischemic stroke without AF (*n* = 37), patients with paroxysmal AF without stroke (*n* = 11), and patients with stroke and new-onset of AF (*n* = 10). Baseline characteristics of study participants and echocardiographic parameters are presented in Table 2. Medication documented at study inclusion is provided in Table S4. Participants in the young control group without AF and stroke were significantly younger. Patients did not receive unfractionated heparin prior to blood sampling. Low molecular heparin was administered for a minimum of 12 h prior to blood sampling. No patient received Vitamin K antagonists and mineralocorticoid receptor antagonists at inclusion to the study. Medication with non-vitamin K antagonist oral anticoagulants, angiotensin-converting enzyme inhibitors, and ß-blockers were significantly frequent in patients with AF. However, in patients with AF hypertension was more frequent in comparison to patients without AF, explaining the wider use of angiotensin-converting enzyme inhibitors and ß-blockers in these patients. No significant difference was observed between patients with and without AF regarding left ventricular ejection fraction. In patients with AF echocardiographic parameters of left atrial function (LAVI/a′, SRa) and septal PA-TDI were significantly altered, compared to participants without AF as provided in Figure 2. Of note, there was no statistical significant difference between patients with and without stroke regarding these parameters. 

### 3.2. Soluble ST−2, Gal3 and NT-proBNP

**Figure 3:**
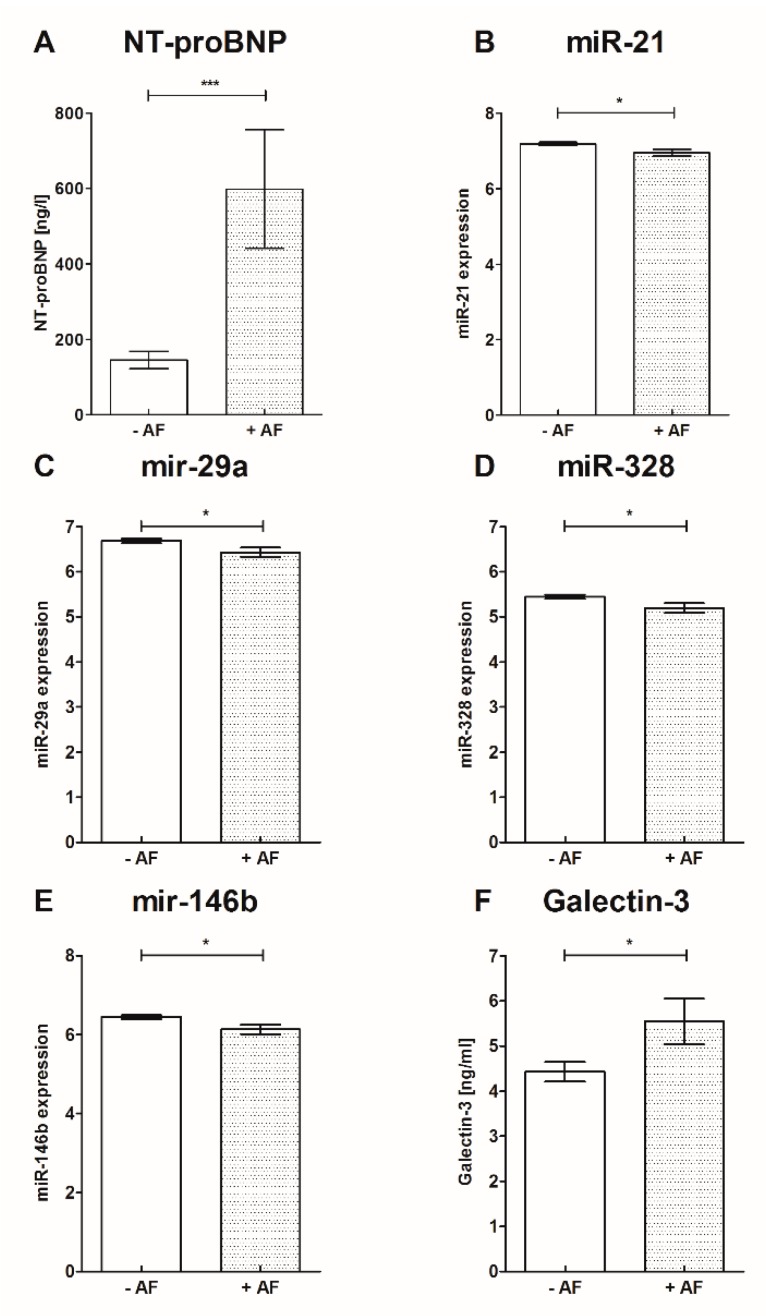
Biomarkers and miRs were altered in patients with and without AF. **A**: NT-proBNP, **B**: miR−21, **C**: miR−29a, **D**: miR−328, **E**: miR−146b, **F**: Galectin−3 mean ± SD; * *p* < 0.05, *** *p* < 0.001; miR- Micro ribonucleic acid, NT-proBNP- N-terminal pro-brain natriuretic peptide.

Results of sST−2, Gal3 and NT-proBNP biomarker analysis are presented in Table 3 and Figure 3: Soluble ST−2, Gal3 and NT-proBNP were significantly increased in patients with AF in comparison to patients without AF. Furthermore, NT-proBNP was significantly increased in patients with AF independent of stroke presence compared to controls. Soluble ST−2 and Gal3 were significantly increased in patients with stroke and AF compared to patients with stroke without AF. Levels of s-ST2 and Gal3 were significantly higher with the presence of stroke (s-ST2 27.70 ± 9.91 vs. 22.33 ± 7.86, *p* = 0.01; Gal3 5.29 ± 2.14 vs. 3.99 ± 1.32, *p* = 0.006). The tested biomarkers did not correlate with the levels of C-reactive protein (s-ST2 r = 0.188, *p* = 0.148; Gal3 r = 0.009, *p* = 0.945; NT-proBNP r = 0.013, *p* = 0.917).

### 3.3. MicroRNA-Panel Results

In our analysis the microRNA panel included miRs which are associated with atrial remodeling (miR−29a, miR−146b), electrical remodeling (miR−328), cardiac fibrosis (miR−21, miR−133a, miR−146b) and atrial fibrillation (miR−21, miR−29a). 

Comparing patients with AF to patients without AF, miR−21, miR−29a, miR−146b and miR−328 were significantly downregulated in patients with AF, while miR−133a was not significantly altered. None of the miRs correlated with the presence of stroke (miR−21 r = 0.014, *p* = 0.90; miR−29a r = 0.052, *p* = 0.65; miR−133a r = 0.084, *p* = 0.49; miR−146b r = 0.005, *p* = 0.97; miR−328 r = 0.038, *p* = 0.74) and the acute-phase protein C-reactive protein (miR−21 r = 0.171, *p* = 0.17; miR−29a r = 0.072, *p* = 0.57; miR−133a r = 0.078, *p* = 0.54; miR−146 r = 0.09, *p* = 0.47; miR−328 r = 0.08, *p* = 0.54). MicroRNA-array results are provided in Table 3 and Figure 3.

### 3.4. Correlation of Biomarkers and Echocardiographic Parameters of Left Atrial (LA) Function and Remodeling

In this study of patients with preserved ejection fraction, miR−21, miR−29a, miR−133a, miR−146b, miR−328, s-ST2, Gal3 and NT-proBNP did not correlate with left ventricular ejection fraction and parameters of diastolic dysfunction assessed by echocardiography. The echocardiographic parameter sPA-TDI, reflecting the septal total atrial conduction time, significantly correlated with serum concentrations of sST2 (r = 0.249, *p* = 0.048), miR−21 (r = −0.277, *p* = 0.012), miR−29a (r = −0.296, *p* = 0.015), miR−146b (r = −0.319, *p* = 0.004), miR−328 (r = −0.296, *p* = 0.008) and NT-proBNP (r = 0.426, *p* =< 0.001). 

**Figure 4:**
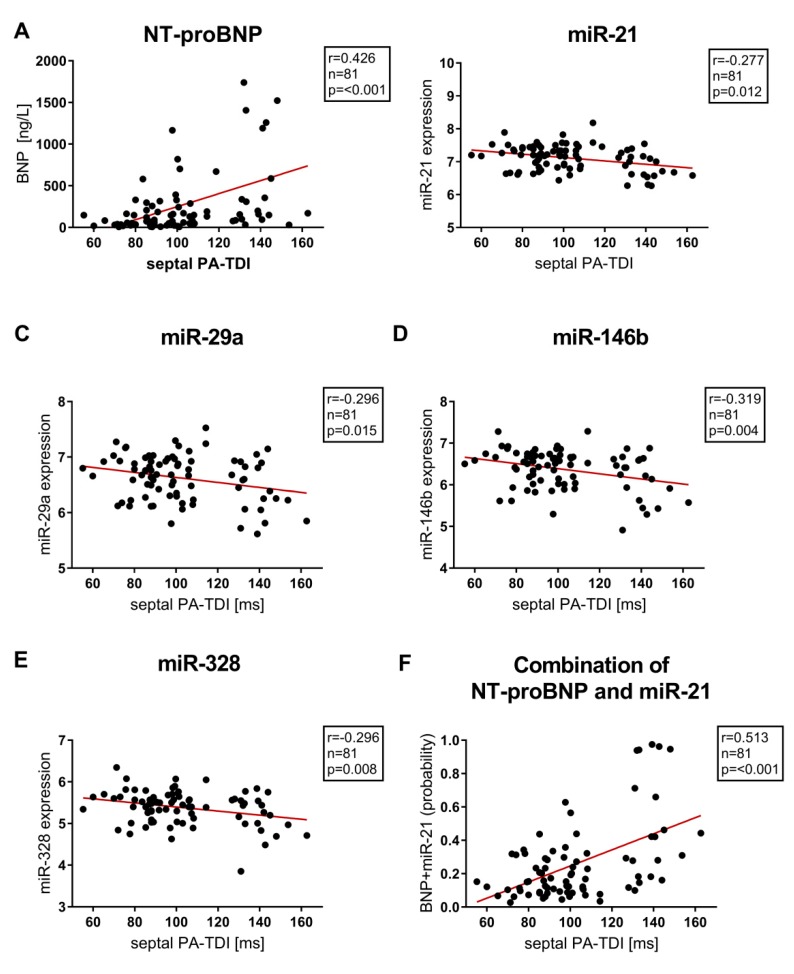
Correlation between septal PA-TDI, NT-proBNP, single micro RNAs (miR−21, miR−29a, miR−146b, miR−328) and combined miR−21 & NT-proBNP to distinguish between patients with and without AF. **A**: NT-proBNP, **B**: miR−21, **C**: miR−29a, **D**: miR−146b, **E**: miR−328, **F**: Combination of NT-proBNP and miR−21 (probability) miR- Micro ribonucleic acid, NT-proBNP- N-terminal pro-brain natriuretic peptide, septal PA-TDI- septal total atrial conduction time.

Furthermore, NT-proBNP correlated highly significantly with additional echocardiographic parameters associated with left atrial remodelling and dysfunction (LAVI/a′ r = 0.457, *p* = 0.001; SRa (second negative peak strain rate during LA contraction) r = 0.462, *p* = 0.001). However, miR−133a was the only miR not correlating with echocardiographic parameters of LA function and remodeling (septal PA-TDI r = −0.153, *p* = 0.172; LAVI/a′ r = 0.07, *p* = 0.52; SRa r = 0.06, *p* = 0.61). Correlation between septal PA-TDI and Biomarkers/miR are provided in Figure 4.

### 3.5. Biomarkers and Prediction of Atrial Fibrillation (AF)

To exclude the influence of statistical outliers and to analyze a prediction model the entire cohort was divided in a random manner into a training and a test set. The prediction model was built in the training model and subsequent confirmed in the test and entire set. 

The predictive validity of NT-proBNP and miR−21 was consistent in regression analysis and the ROC analysis of the training and test set. Regression analysis and ROC-analysis of the training and test set are provided in Tables S1 and S2.

Univariate regression analysis of the complete cohort showed Gal3, miR−21, miR−29a, miR−146b, miR−328 and NT-proBNP as predictors of AF. However, multivariable regression analysis highlighted miR−21 (HR 0.16 (95%CI 0.04–0.7), *p* = 0.009) and NT-proBNP (HR 1.002 (95%CI 1.001–1.004), *p* = 0.006) as independent predictors with the presence of AF in the complete cohort (as provided in Table 4).

The discriminative ability of miR−21, NT-proBNP and the combination of miR−21 and NT-proBNP were verified by the McNemar test. Upon acceptance of a dichotomous distribution, McNemar test revealed a better specificity for combination of NT-proBNP and miR−21 compared to NT-proBNP and miR−21. Furthermore, the combination of NT-proBNP and miR−21 had a comparable sensitivity to NT-proBNP and a better sensitivity in comparison to miR−21. Results from the McNemar test are provided in Table S3. The combination of NT-proBNP and miR−21 correlated strongly with septal PA-TDI (Figure 4F).

## 4. Discussion

In our current prospective study we analyzed different circulating miRs and biomarkers involved in atrial remodeling and fibrosis to identify specific blood-based biomarkers predicting AF. 

Clinically silent AF often is underdiagnosed and screening of patients at risk seems to be a meaningful strategy [34]. Prolonged rhythm monitoring extending even beyond 72 h ECG-monitoring as recommended by current AF ESC guidelines in patients after stroke has been suggested [3,34,40]. However, prolonged cardiac rhythm monitoring for detection of subclinical AF requires significant diagnostic resources and potentially affects patients’ integrity as well as compliance. Therefore, predictors of subclinical AF in patients at risk and patients after stroke would be important as potential screening markers to detect patients with high chances for the presence of subclinical AF, which can be further evaluated with risk-adapted rhythm monitoring.

In the present study, we demonstrated in a cohort with sinus rhythm, preserved ejection fraction and without myocardial infarction that miR−21, miR−29a, miR−146b and miR−328 were significantly decreased in patients with clinical silent AF in comparison to patients without AF. In contrast, s-ST2, Gal−3 and NT-proBNP were significantly increased in patients with diagnosed AF. Multivariable regression analysis in a test and in a training set showed the predictive value of miR−21 and NT-proBNP. Furthermore, in the entire set multivariable regression analysis highlighted miR−21 and NT-proBNP as independent predictors of AF. 

In patients with heart failure based on impaired systolic or diastolic ventricular function NT-proBNP, Gal3 and s-ST2 are associated with poor clinical outcome [20,21,41]. In contrast, in patients with AF and without structural heart disease circulating NT-proBNP is increased even in the presence of sinus rhythm [42,43]. However, NT-proBNP is associated with the presence and burden of AF [44]. After ablation of AF, NT-proBNP is a predictor of AF relapse [45]. Gal3 conciliates fibrotic pathways and is associated with detrimental ventricular and atrial remodeling [23,46,47]. In addition, the presence of AF is associated with elevated Gal3 plasma levels [48]. This is in line with our findings showing elevation of sST2, Gal3 and NT-proBNP in patients with AF. 

Previous studies depicted miR−21 as a regulator of cardiac fibrosis. Inhibition of miR−21 suppresses fibrosis in mice after transverse aortic constriction and reduces AF duration in rats after initiation of myocardial infarction [26,49]. In patients with AF a decrease of circulating miR−21 was shown [50,51]. Thus, our results validate these findings. In the present study miR−29, miR−146b and miR 328 were significantly downregulated in patients with AF. miR−29 is potentially associated with collagen regulation of the atrium. In the study of Dawson et al., circulating miR−29 levels were also decreased in patients with AF [30]. Adverse electrical remodeling in an experimental model with AF in dogs was associated with levels of miR−328 [52]. Additionally, mal-structural remodeling by fibrosis is probably mediated via TIMP−4 by miR−146b [53]. miR−133 has pro-fibrotic properties mediated by inhibition of connective tissue growth factor [54]. However, in the present study the expression of circulating miR−133 was not significantly altered in patients with AF. Besides, in patients with paroxysmal, symptomatic AF unresponsive to medical treatment effective AF ablation is associated with a favorable reverse atrial remodeling and increased expression of miR-21- a regulator of atrial fibrosis-, miR-150-associated with fibrosis and remodeling-, and miR-409-associated with the progression of AF burden-, representing potential targets to prevent relapse after AF ablation approach [55].

Subsequently, we correlate the tested biomarkers to echocardiographic parameters. LA-remodeling and fibrosis are well established arrhythmic substrates, which correlate to echocardiographic parameters of LA function and remodeling [13]. A ratio of indexed LA volume and mitral annulus velocity during atrial contraction (LAVI/a′) express atrial ejection function and is a predictor of AF [11,14,16].

Additionally, left atrial strain and left atrial strain rate assessed by 2D speckle-tracking echocardiography facilitate evaluation of regional LA-dysfunction and fibrosis [13,56]. The strain rate parameter SRa assessed during the contractile phase of the LA is associated with AF [16,57]. However, besides adding additional diagnostic value, speckle trecking analysis of the LA involves some methodological complexities: Implementation of LA strain is challenging, as the left atrial wall is thin compared with the wall of the LV, making adequate tracking more difficult. Furthermore, LA strain is directly influenced by the LV filling pressures and, therefore, by the LV function, leading to a high variability of the measurements [58]. Another important limitation of LA strain is its lack of standardization, leading to a broad range of the normal reference [59]. This lack of a clear consensus on the normal reference ranges for left atrial strain makes it difficult to interpret in clinical routine. However, in the present monocentric study, determination of LA strain in our hands was performed in a standardized manner. Therefore, we think LA strain data are of significant value for interpretation in the context of the overall data set in this selected patient population. Furthermore, total atrial conduction time, determined at the lateral mitral valve ring, has been shown to correlate with the extent of left atrial fibrosis [15]. Moreover, a recent published echocardiographic parameter, septal PA-TDI, seems to be a predictor of subclinical AF and a promising parameter for risk-stratified reduction of ECG monitoring duration after stroke [16].

Gal3 and miR−133a were not associated with echocardiographic parameters. Actually, in the present study echocardiographic parameters reflecting LA dysfunction and remodeling correlate with levels of sST2, miR−21, miR−29a, miR−146b, miR−328 and NT-proBNP. In particular, septal PA-TDI correlates with sST2, miR−21, miR−29a, miR−146b, miR−328 and NT-proBNP, which are connected to fibrotic changes and associated with atrial remodeling. Hence, these finding might account for potential association of septal PA-TDI with atrial fibrosis. However, the intra- and extracellular effects of miRs, their origin as well as their metabolism are not entirely investigated. Therefore, our results do not provide a conclusion of tissue-specific release of miRs and pathophysiological background of septal PA-TDI.

Furthermore, in our study the combination of NT-proBNP and miR−21 was associated with a preferable accuracy in predicting AF. Multivariate analysis depicted the combination of NT-proBNP and miR−21 as the most powerful predictor of AF in our study. The discriminative ability was enhanced by the combination of both biomarkers as revealed in ROC curve analysis (AUC = 0.84) compared to NT-proBNP (AUC = 0.76) and miR−21 (AUC = 0.30) alone.

To our knowledge, no other study compared the predictive value of NT-proBNP, s-ST2, Gal3 and miRs for AF in patients with and without stroke. Furthermore, we described for the first time the potential association of biomarkers involved in atrial fibrosis and the echocardiographic parameter septal PA-TDI, which seemed to be a strong predictor of AF. Especially, after AF-ablation NT-proBNP, miR-21 and septal PA-TDI may be promising to investigate the therapy-response and to allocate patients to new monitoring systems to avoid AF recurrence [60]. In everyday clinical practice the identified biomarkers – in particular NT-pro-BNP - could be used to screen patients at risk for AF (e.g., after embolic stroke of undetermined source). Subsequent patients with elevated biomarker levels should be allocated to a comprehensive echocardiographic evaluation including septal PA-TDI for further risk stratification. The clinical value of this workflow should be tested in a prospective study.

In this study limitations should be considered: (1) This study included a small sample size within different groups. However, a detailed patient characterization with a rigorous diagnostic process with a complete echocardiographic protocol, ECG-monitoring and blood sample acquisition, the prospective design, and the inclusion of patients with and without stroke strengthen this study. Therefore, the predictive performance of NT-proBNP and miR-21 can be applied in both cohorts. (2) The present study is only descriptive and cannot contribute to an understanding of the pathomechanisms behind the regulated miR and biomarkers in AF. (3) A potential selection bias owing to biomarker and miR selection has to be considered. However, it should be recognized that other miR and biomarkers may also display predictive properties in AF. 

## 5. Conclusions

This study confirmed the potential role of biomarkers for the prediction of AF. We demonstrated that biomarkers and especially miRs are associated with AF and correlate with echocardiographic parameters reflecting LA function and remodeling. In particular, NT-proBNP and miR−21 are independent predictors of AF in this study. The combination of both enhances the ability to discriminate between patients with and without AF. Thus, miR−21 and NT-proBNP are candidate AF-biomarkers for additive risk-stratified decision-making. Further studies in greater scale are warranted to verify this hypothesis.

## Figures and Tables

**Figure 5:**
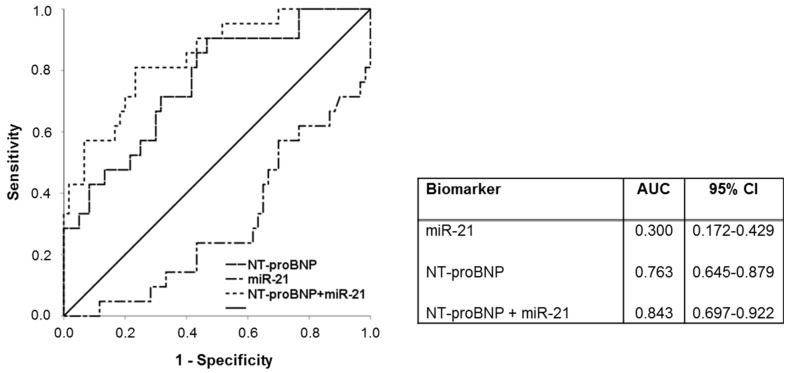
ROC-curve analysis of NT-proBNP, miR−21 and combined NT-proBNP and miR−21. miR- Micro ribonucleic acid, NT-proBNP- N-terminal pro-brain natriuretic peptide.

**Table 1:** MicroRNA primer sequences.

microRNA	Sequence
hsa-miR−21−5p	UAGCUUAUCAGACUGAUGUUGA
hsa-miR−29a−3p	UAGCACCAUCUGAAAUCGGUUA
hsa-miR−133a−3p	UUUGGUCCCCUUCAACCAGCUG
hsa-miR−146b−5p	UGAGAACUGAAUUCCAUAGGCUG
hsa-miR−328−3p	CUGGCCCUCUCUGCCCUUCCGU
hsa-miR−486−5p	UCCUGUACUGAGCUGCCCCGAG
Cel-miR−39−3p	UCACCGGGUGUAAAUCAGCUUG

**Table 2:** Patient characteristics and echocardiographic parameters.

Parameter		All Patients			w/o Stroke			Stroke	
		−AF (*n* = 60)	+AF (*n* = 21)	*p* −AF vs. +AF	−AF, Young (*n* = 13)	−AF, Old (*n* = 10)	+AF (*n* = 11)	−AF (*n* = 37)	+AF (*n* = 10)
Age (years)		58.2 ± 19	71.8 ± 11.3	0.005	30.1 ± 6.6	65.5 ± 10.3 ^#^	67.7 ± 12.7 ^#^	65.8 ± 13.9 ^$^	76.2 ± 8
Male		42 (70%)	14 (66.7%)	0.853	10 (76.9%)	6 (60%)	9 (81.8%)	26 (70.3%)	5 (50%)
Height (cm)		173.5 ± 9.6	170.6 ± 10.7	0.246	180.5 ± 6.5	171.3 ± 8.9	173.1 ± 11.01	171.7 ± 9.7	167.8 ± 10.2
Weight (kg)		79.3 ± 13.2	76.1 ± 16.6	0.667	78.1 ± 10.2	78.4 ± 14.3	79 ± 18.1	79.6 ± 14.4	72.9 ± 15.0
**Pre-existing Conditions**									
	Hypertension	31 (50.8%)	17 (81.0%)	0.016	0 (0.0%)	3 (30%)	8 (72.7%) ^##^	26 (70.3%)	9 (90%)
	Diabetes	11 (18.0%)	5 (23.8%)	0.565	0 (0.0%)	1 (10%)	0 (0.0%)	10 (27%)	4 (40%)
	Smoking	13 (21.3%)	4 (19.0%)	0.852	1 (7.7%)	3 (30%)	3 (27.3%)	9 (24.3%)	1 (10%)
	Stroke	13 (21.3%)	4 (19.0%)	0.811	0 (0.0%)	0 (0.0%)	0 (0.0%)	11 (29.7%)	3 (30%)
	CKI	5 (8.2%)	1 (4.8%)	0.602	0 (0.0%)	0 (0.0%)	0 (0.0%)	5 (13.5%)	1 (10%)
	CAD	9 (14.8%)	6 (28.6%)	0.158	0 (0.0%)	2 (20%)	3 (27.3%)	7 (18.9%)	3 (30%)
	PAD	5 (8.2%)	2 (9.5%)	0.851	0 (0.0%)	1 (10%)	2 (18.2)	4 (10.8%)	0 (0.0%)
**Echocardiographic Parameters**									
LVEF (%)		60.3 ± 4.9	59.4 ± 7.66	0.840	61.2 ± 2.7	59.0 ± 6.8	62.1 ± 6.2	60.4 ± 5.0	58.9 ± 8.3
PA-TDI septal (ms)		90.8 ± 13.6	138.8 ± 8.9	<0.001	85.5 ± 13.2	92.7 ± 17.7	140.4 ± 11.2 ^###§§§^	91.8 ± 12.7 ^$$$^	137 ± 5.5
PA-TDI lateral (ms)		101.6 ± 11.7	149.2 ± 13.7	<0.001	97.3 ± 9	101.7 ± 9.9	154.5 ± 16.2 ^###§§§^	102.5 ± 12.8 ^$$$^	143.4 ± 7.6
LAVI/a′		3.1 ± 0.9	5.7 ± 3.4	<0.001	3.7 ± 0.6	3.1 ± 1.2	4.3 ± 1.5 ^§^	3.0 ± 0.9 ^$$$^	7.2 ± 4.3
SRa (s^−1)^		−2.1 ± 0.7	−1.3 ± 0.6	<0.001	−1.9 ± 0.4	−2.0 ± 1.0	−1.5 ± 0.7	−2.3 ± 0.7 ^$$$^	−1.1 ± 0.3

AF—Atrial fibrillation, CAD—Coronary artery disease, CKI—Chronic kidney disease, LAVI—Left atrial volume index, LVEF—Left ventricular ejection fraction, PAD—Peripheral artery disease, PA-TDI—Total atrial conduction time, SRa—Second negative peak strain rate during left atrial contraction. ^#^ *p* < 0.05 vs. young −AF w/o stroke, ^##^ *p* < 0.01 vs. young −AF w/o stroke, ^###^ *p* < 0.001 vs. young −AF w/o stroke, ^§^ *p* < 0.05 vs. old −AF w/o stroke, ^§§§^ *p* < 0.001 vs. old −AF w/o stroke, ^$^ *p* < 0.05 vs. stroke +AF, ^$$$^ *p* < 0.001 vs. stroke +AF.

**Table 3:** Biomarkers.

Parameter	All Patients			w/o Stroke			Stroke	
	−AF (*n* = 60)	+AF (*n* = 21)	*p* −AF vs. +AF	−AF, Young (*n* = 13)	−AF, Old (*n* = 10)	+AF (*n* = 11)	−AF (*n*= 37)	+AF (*n* = 10)
s-ST2 (ng/mL)	24.9 ± 12.8	29.3 ± 10.1	0.038	21 ± 7.12	22.1 ± 8.3	26 ± 9.3	24.7 ± 9 ^$^	31.6 ± 11.4
Galectin−3 [ng/mL]	4.4 ± 1.7	5.5 ± 2.3	0.015	3.8 ± 1.3	4.3 ± 1.4	4.4 ± 1.3	4.6 ± 1.7 ^$^	6.4 ± 1.7
miR−21	7.2 ± 0.4	7 ± 0.4	0.013	7.2 ± 0.3 *	7.2 ± 0.4	6.9 ± 0.3	7.2 ± 0.4	7.0 ± 0.4
miR−29a	6.7 ± 0.4	6.4 ± 0.5	0.015	6.7 ± 0.4	6.7 ± 0.4	6.4 ± 0.4	6.7 ± 0.3	6.4 ± 0.3
miR−133a	5.7 ± 0.5	5.6 ± 0.6	0.389	5.9 ± 0.5	5.8 ± 0.5	5.5 ± 0.5	5.7 ± 0.5	5.6 ± 0.7
miR−146b	6.5 ± 0.4	6.1 ± 0.6	0.026	6.6 ± 0.4 **	6.3 ± 0.4	6.1 ± 0.5	6.4 ± 0.4	6.2 ± 0.6
miR−328	5.5 ± 0.4	5.2 ± 0.5	0.014	5.5 ± 0.3 *	5.3 ± 0.2	5.1 ± 0.4	5.4 ± 0.4	5.2 ± 0.6
NT-proBNP (ng/L)	75.7 (34.7–198.9)	199.6 (96.2–1225)	<0.001	35.9 ± 24.9	90 (35.1–215)	156.1 (77.8–1190) ^##^	139.3 (49.1–302.6) ^$^	346.6 (156.5–1489)

miR-Micro ribonucleic acid, NT-proBNP-N-terminal pro-brain natriuretic peptide, s-ST2- soluble ST2, ST2- suppression of tumorigenicity 2. * *p* < 0.05 vs. +AF w/o stroke, ** *p* < 0.01 vs. +AF w/o stroke, ^##^ *p* < 0.01 vs. −AF, young w/o stroke, ^$^ *p* < 0.05 vs. stroke +AF.

**Table 4:** Biomarker predicting AF in complete cohort (∑*n* = 81, AF *n* = 21).

Parameter	Univariate Regression Analysis		Multivariate Regression Analysis	
	Hazard Ratio (95% Confidence Interval)	*p*	Hazard Ratio (95% Confidence Interval)	*p*
s-ST2 (ng/mL)	1.03 (0.99–1.07)	0.179		
Galectin−3 (ng/mL)	1.33 (1.02–1.73)	**0.04**	1.20 (0.86–1.67)	0.27
miR−21	0.17 (0.04–0.74)	**0.02**	0.16 (0.04–0.7)	**0.009**
miR−29a	0.22 (0.06–0.78)	**0.02**	1.16 (0.03–52−2)	0.941
miR−133a	0.55 (0.2–1.45)	0.23		
miR−146b	0.25 (0.08–0.74)	**0.01**	0.71 (0.01–64.3)	0.71
miR−328	0.21 (0.06–0.8)	**0.02**	1.33 (0.09–20.7)	0.44
NT-proBNP (ng/L)	1.002 (1.001–1.004)	**0.003**	1.002 (1.001–1.004)	**0.006**

miR—Micro ribonucleic acid, NTproBNP—N-terminal pro-brain natriuretic peptide, s-ST2—soluble ST2, ST2—suppression of tumorigenicity 2. Receiver operating characteristic (ROC) curve analysis presenting the predictive ability of miR−21, NT-proBNP and the combination of NT-proBNP and miR−21, are presented in Figure 5. Of note, the AUC is clearly enhanced by addition of miR−21 to NT-proBNP (AUC = 0.843) in comparison to NT-proBNP (AUC = 0.763) and miR−21 (AUC = 0.300) alone.

## Supplementary Materials

The following are available online at https://www.mdpi.com/2077-0383/9/4/1118/s1, Table S1: Regression analysis of training and test set, Table S2: ROC-analysis of training and test set, Table S3: Mc Nemar test for dependent samples, Table S4: Medication documented at study inclusion.

## Abbreviations

AF	Atrial fibrillation
Cis	Confidence intervals
Gal3	Galectin−3
HR	Hazard ratio
LA	Left atrial
LAVI/á	Ration of LA volume index to tissue Doppler á
miR	MicroRNA
NT-proBNP	N-terminal fragment of pro-B-type natriuretic peptide
PA-TDI	Total atrial conduction time
ROC	Receiver operating characteristic
SRa	Second negative peak strain rate during LA contraction
sST2	Soluble suppression of tumorigenicity 2
