# Supplementary material for: miR−21 and NT-proBNP Correlate with Echocardiographic Parameters of Atrial Dysfunction and Predict Atrial Fibrillation
Source: J Clin Med. 2020 Apr 14;9(4):1118. doi: 10.3390/jcm9041118 (PMC7230176; doi:10.3390/jcm9041118)
Supplement: Supplementary file 1 [file jcm-09-01118-s001.pdf]

Table S1. Regression analysis of training and test set.

	Training Set (∑n = 35, AF n = 11)				Test Set (∑n=46, AFn = 10)			
**Parameter**	**Univariate regression analysis**		**Multivariate regression analysis**		**Univariate regression analysis**		**Multivariate regression analysis**	
	**HR (CI 95%)**	**P**	**HR (CI 95%)**	**P**	**HR (CI 95%)**	**P**	**HR (CI 95%)**	**P**
s-ST2 (ng/ml)	1.09 (0.99–1.21)	**0.093**	1.07 (0.05–1.21)	0.277	1.06 (0.99–1.13)	0.117		
Gal3 (ng/ml)	1.47 (0.88–2.45)	0.141			1.30 (0.94–1.77)	0.111		
miR-21	0.05 (0.003–0.782)	**0.033**	0.02 (0.001–0.78)	**0.036**	0.16 (0.03–0.99)	**0.049**	0.15 (0.02–1.12)	0.064
miR-29a	0.11 (0.01–0.92)	**0.042**	0.83 (0.001–19119.53)	0.88	0.39 (0.074–2.10)	0.271		
miR-133a	0.71 (0.15–3.37)	0.666			0.40 (0.11–1.50)	0.175		
miR-146b	0.11 (0.01–0.80	**0.029**	1.81 (0.001–5727.67)	0.886	(0.39 (0.11–1.52)	0.178		
miR-328	0.07 (0.006–0.88)	**0.039**	5.2 (0.02–1433.12)	0.57	0.35 (0.07–1.65)	0.185		
NT-proBNP (ng/L)	1.002 (1.00–1.004)	**0.02**	1.002 (1.00–1.005)	**0.042**	1.003 (1.00–1.006)	**0.093**	1.002 (1.00–1.004)	0.098

Table S2. ROC-analysis of training and test set

	Training Set (∑*n*= 35, AF *n* = 11)		Test Set (∑*n* = 47, AF *n* = 10)	
**Parameter**	**AUC**	**95% CI**	**AUC**	**95% CI**
s-ST2 [ng/ml]	0.686	0.487–0.886		
Gal3 [ng/ml]				
miR-21	0.232	0.061–0.402	0.315	0.122–0.508
miR-29a	0.264	0.069–0.458		
miR-133a				
miR-146b	0.282	0.082–0.482		
miR-328	0.264	0.080–0.448		
NT-proBNP [ng/L]	0.773	0.596–0.949	0.732	0.564–0.901

Table S3. Mc Nemar test for dependent samples

**Biomarker**	**Patients – AF (n=60)**			**Patients + AF (n=21)**		
	**NT-proBNP**	**miR-21**	**NT-proBNP+miR21**	**NTproBNP**	**miR-21**	**NT-proBNP+miR21**
**NT-proBNP**		0.122	0.016		<0.001	1.00
**miR-21**	0.122		<0.001	<0.001		0.004
**NT-proBNP + miR21**	0.016	<0.001		1.00	0.004	

Table S4. Medication documented at study inclusion

Parameter	All patients -AF (*n* = 60)	+ AF (*n* = 21)	P –AF vs +AF
Vitamin K antagonists	0	0	
NOAC	2 (3.3%)	11 (52.4%)	<0.001
ASA	28 (46.7%)	4 (19%)	0.026
Statine	24 (40%)	12 (57.1%)	0.174
ACE-inhibitors	14 (23.3%)	11 (52.4%)	0.013
ARB	14 (23.3%)	5 (23.8%)	0.965
ß-blockers	11 (18.3%)	15 (71.4%)	<0.001
MRA	0	0	

ACE-inhibitors- Angiotensin-converting-enzyme inhibitors, ARB- Angiotensin II receptor blockers, ASA- Acetylsalicylic acid, MRA- Mineralocorticoid receptor antagonist, NOAC- Non-vitamin K antagonist oral anticoagulants
